# Delineating transcriptional crosstalk between *Mycobacterium avium* subsp. *paratuberculosis* and human THP-1 cells at the early stage of infection via dual RNA-seq analysis

**DOI:** 10.1186/s13567-022-01089-y

**Published:** 2022-09-13

**Authors:** Hong-Tae Park, Sang-Mok Lee, Seyoung Ko, Suji Kim, Hyun-Eui Park, Min-Kyoung Shin, Donghyuk Kim, Han Sang Yoo

**Affiliations:** 1 Department of Infectious Diseases, College of Veterinary Medicine, Seoul National University, Seoul, 08826 Korea; 2 School of Energy and Chemical Engineering, Ulsan National Institute of Science and Technology (UNIST), Ulsan, 44919 Korea; 3 Department of Microbiology, College of Medicine, Research Institute of Life Science, Gyeongsang National University, Jinju, 52828 Korea

**Keywords:** *Mycobacterium avium* subsp. *paratuberculosis*, dual RNA-seq, transcriptome, host–pathogen interactome, metabolism

## Abstract

*Mycobacterium avium* subsp. paratuberculosis (MAP) is the causative agent of Johne’s disease, a chronic debilitating disease in ruminants. To control this disease, it is crucial to understand immune evasion and the mechanism of persistence by analyzing the early phase interplays of the intracellular pathogens and their hosts. In the present study, host–pathogen interactions at the transcriptomic level were investigated in an in vitro macrophage infection model. When differentiated human THP-1 cells were infected with MAP, the expression of various genes associated with stress responses and metabolism was altered in both host and MAP at 3 h post-infection. MAP upregulates stress-responsive global gene regulators, such as two-component systems and sigma factors, in response to oxidative and cell wall stress. Downstream genes involved in type VII secretion systems, cell wall synthesis (polyketide biosynthesis proteins), and iron uptake were changed in response to the intracellular environment of macrophages. On the host side, upregulation of inflammatory cytokine genes was observed along with pattern recognition receptor genes. Notably, alterations in gene sets involved in arginine metabolism were observed in both the host and MAP, along with significant downregulation of *NOS2* expression. These observations suggest that the utilization of metabolites such as arginine by intracellular MAP might affect host NO production. Our dual RNA-seq data can provide novel insights by capturing the global transcriptome with higher resolution, especially in MAP, thus enabling a more systematic understanding of host–pathogen interactions.

**Supplementary Information:**

The online version contains supplementary material available at 10.1186/s13567-022-01089-y.

## Introduction

*Mycobacterium avium* subsp. paratuberculosis (MAP) is a causative agent of paratuberculosis (PTB), more commonly known as Johne’s disease (JD), which is a chronic and debilitating disease in ruminants. MAP is also considered to be a potential cause of human Crohn’s disease (CD) because MAP is associated with CD [1, 2].

To understand the pathogenic mechanisms of MAP infection, it is important to elucidate the within-host dynamics of MAP, especially during the initial stage of infection. Similar to other mycobacteria, MAP survives in host macrophages by inhibiting phagolysosomal maturation [3, 4]. Inside the macrophage, the bacterium is not eliminated by the host immune response, and as a result, granulomas are formed in the host, and infection continues in the form of latent infection. When MAP infects immune cells such as macrophages, inflammatory cytokines such as IL-1β and various chemokines are expressed during infection [5, 6]. In particular, several cytokines involved in the differentiation and maintenance of IL-17-producing T cells, such as IL-23, CCL3, CCL4, and CCL5, are upregulated in MAP-infected human THP-1 cells, as shown in our previous study and also in other studies [7–9]. A series of reactions occur through the activation of specific transcription factors of antigens of MAP, such as mannosylated lipoarabinomannan (ManLAM), to activate pattern recognition receptors [10]. Through this, it was possible to understand various host factors in terms of the immune response to MAP infection and its persistence. Additionally, it was suggested that the Th17 inducible immune response, which is considered to be important in chronic infection, is related to subsequent granuloma formation [11–13]. However, since the analysis of host–pathogen interactions in the early stage of infection has mainly focused on the observation of host cell responses, there are few studies on how MAP reacts to the host physiological responses. Few attempts have been made to explain host–pathogen interactions during MAP infection in terms of stress responses and energy metabolism.

Comparative analysis at the genome level has been actively conducted as a strategy to understand the pathogenesis of MAP. Thanks to the recently developed whole genome sequencing technology, additional genome information has become available for many isolates of MAP, and extensive comparative analyses have been performed on functional units of potential virulence factors through pangenome-based analysis [14, 15]. Through these studies, it was confirmed that MAP has a highly conserved genome compared to other subspecies, such as *M. avium* subsp. *hominissuis* (Mah), and it shares common virulence genes between MAP strains [14, 15]. Comparative genomic analysis at the DNA level has greatly helped in discovering potential virulence factors, but confirmation at the RNA level is necessary to ensure that they actually perform the expected functions in the infectious environment.

To date, dual RNA-seq is one of the most effective tools for understanding host–pathogen interactions [16]. It is useful for investigating global changes in gene expression, especially in intracellular pathogens, because a “snapshot” can be taken of both the host and microbe by extracting their RNA simultaneously. In this study, we infected THP-1 cells, a human monocyte derived cell line, with MAP to investigate the global gene expression changes in both organisms. Despite the controversy over whether THP-1 is an appropriate alternative source for its physiological counterpart, human peripheral blood mononuclear cells, particularly in terms of macrophage polarization [17, 18], we believed that this infection model is suitable for determining the initial interaction because the infectivity of MAP to THP-1 has been confirmed in our previous study along with the gene expression changes in infected THP-1 cells [9]. By performing dual RNA-seq analysis after 3 h of infection of THP-1 cells with MAP, the differentially expressed genes (DEGs) of MAP were enriched by using the KEGG pathway database, as well as by BLAST analysis of the MAP genome against the virulence factor database (VFDB). Our data showed that MAP expressed stress-responsive genes, and metabolism-related genes were significantly changed in both the host and MAP. The results also showed that the utilization of specific metabolites, such as arginine, might affect host defense mechanisms, such as NO production. Overall, these stress-induced global changes in virulence-related gene expression contribute to revealing the mechanisms for survival and persistence of MAP infection, and the related genes can be used for further phenotypic analysis through mutagenesis.

## Materials and methods

### Cell culture, infection and total RNA preparation

Human monocytic THP-1 cells were cultured in RPMI 1640 medium supplemented with 10% heat-inactivated FBS (Gibco) and 1% penicillin/streptomycin at 37 °C with 5% CO_2_. The cells were then differentiated into macrophages by stimulation with 50 ng/mL phorbol 12-myristate 13-acetate (PMA) (Sigma Aldrich) for 72 h of incubation. Differentiated cells were washed twice with FBS-free RPMI 1640 medium and incubated with 5% FBS-RPMI 1640 medium without antibiotics for 24 h before infection.

MAP strain K-10 was prepared for infection. Bacteria were grown at 37 °C in Middlebrook 7H9 broth (Beckton Dickinson) supplemented with Mycobactin J (2 mg/L, Allied Monitor), 0.04% casitone, 0.2% glycerol and 10% oleic acid-albumin-dextrose-catalase enrichment (OADC) for 4 weeks. Then, the bacteria-containing broth media was passed through a 23 gauge needle to minimize clumps. The upper part of the medium was used for subsequent experiments. A total of 5 × 10^6^ THP-1 cells were plated in 75 T cell culture flasks and infected with MAP strain K-10 at a multiplicity of infection (MOI) of 10:1 for 3 h. After 3 h of incubation, the supernatant was discarded and the cells were washed twice with 1 × DPBS to remove noninternalized bacteria.

Total RNA extraction was performed using an RNeasy Mini Kit (Qiagen) following the manufacturer’s instructions with modifications. Because MAP yielded low amounts of RNA, causing an extremely low ratio to host RNA, bacterial RNA was enriched by eliminating most of the host RNA using centrifugation of the total cell lysate. Briefly, infected cells were lysed in 10 mL RLT buffer supplemented with 1% β-mercaptoethanol. Lysed cells were centrifuged at 3000×*g* for 15 min to pellet unlysed MAP cells. Then, 9 mL of the supernatant was discarded, and the pellet was resuspended in 1 mL of the remaining RLT buffer. Total RNA was then extracted using mechanical disruption with 0.1 mm zirconia/silica beads.

### Dual RNA-seq

Library construction for the dual RNA-seq was performed with 1 µg of total RNA for each sample using an Illumina TruSeq Stranded Total RNA Library Prep Human/Mouse/Rat Kit (Illumina, Inc., San Diego, CA, USA). Briefly, removal of rRNA from the total RNA was conducted using the Ribo-Zero rRNA Removal Kit (Human/Mouse/Rat) (Illumina, Inc.) and the NEBNext rRNA Depletion kit (Bacteria) (NEB). After the depletion of the rRNA, the remaining RNA was fragmented into small pieces. The cleaved RNA fragments were copied into first-strand cDNA using SuperScript II reverse transcriptase (Invitrogen) and random primers, followed by second-strand cDNA synthesis using DNA Polymerase I, RNase H and dUTP. After the ligation of adapters to the cDNA, the products were purified and enriched with PCR. The prepared libraries were submitted to Illumina NovaSeq (Illumina, Inc.), and paired-end (2 × 101 bp) sequencing was performed by Macrogen Incorporated.

For the processing of the human transcriptome profile, the relative abundances of genes were measured in Read Count using StringTie. Statistical analysis was performed to find DEGs using the estimates of abundances for each gene in samples. Data were then log_2_-transformed and subjected to TMM normalization. Statistical significance of the differential expression data was determined using exactTest using edgeR and fold change, in which the null hypothesis was that no difference exists among groups. The false discovery rate (FDR) was controlled by adjusting the *p* value using the Benjamini–Hochberg algorithm. For the processing of the bacterial transcriptome profile, sequence reads were mapped onto the reference genome of MAP (NC_002944.2) using Bowtie (v1.2.2) with a maximum insert size of 1000 bp and two maximum mismatches after trimming 3 bp from the 3′ ends [19]. Subsequently, SAM output files generated from Bowtie were transformed to BAM format using SAMtools [20]. The differential gene expression was analyzed using the DESeq2 R package [21]. Among the 4539 genes in the MAP genome, we filtered out 300 genes that were expressed at a level of less than 10 mapped reads from the sum of all samples. Genes with expression values with log_2_-fold change ≥ 1.0 and adjusted *p* value ≤ 0.05 or log_2_ (fold change) ≤ −1.0 and adjusted *p* value ≤ 0.05 were defined as differentially expressed genes (DEGs). The adjusted *p* values were all Benjamini–Hochberg corrected *p* values.

### Data processing

#### Characterization of virulence factors in the MAP genome

To determine virulence factors (VFs) across the MAP genome (NC_002944.2), each locus tag of MAP was queried against VFDB [22], an open resource collecting information about the pathogenomic compositions of various bacteria, to conduct BLASTp analysis (E-value: 1e–10). Subsequently, locus tags were classified by the functional category of their best BLAST hit virulence factor identifier (VFG). Additional functional categorization based on COG categories and KEGG pathways was also conducted, and differentially expressed gene (DEG) information was integrated to identify which VFs in the MAP genome change specifically in the event of infection of the THP-1-cell line.

#### Functional enrichment analysis

Functional enrichment analysis of both humans and bacteria was performed using the GSEA tool (v4.1.0, Broad Institute). The KEGG database from MSigDB (v7.4) was used for the GSEA of the host, and the level C gene set of MAP extracted from the KEGG database was listed and used for the analysis of bacteria. Analysis was conducted with default settings (1000 permutations, weighted statistics, min and max gene-set size 15–500). Enriched pathways of bacteria (*p*  < 0.5) were exported to Cytoscape (v3.9.0) and grouped by EnrichmentMap application [23, 24].

### Statistical analysis

The two-sided Fisher’s exact enrichment was tested on each metabolism of virulence factors using the Python *scipy.stats.fisher_exact* function (SciPy v.1.7.3.). Significance was assumed at *p* < 0.05.

## Results

### Dual RNA-seq of MAP-infected THP-1 macrophages

**Figure 1:**
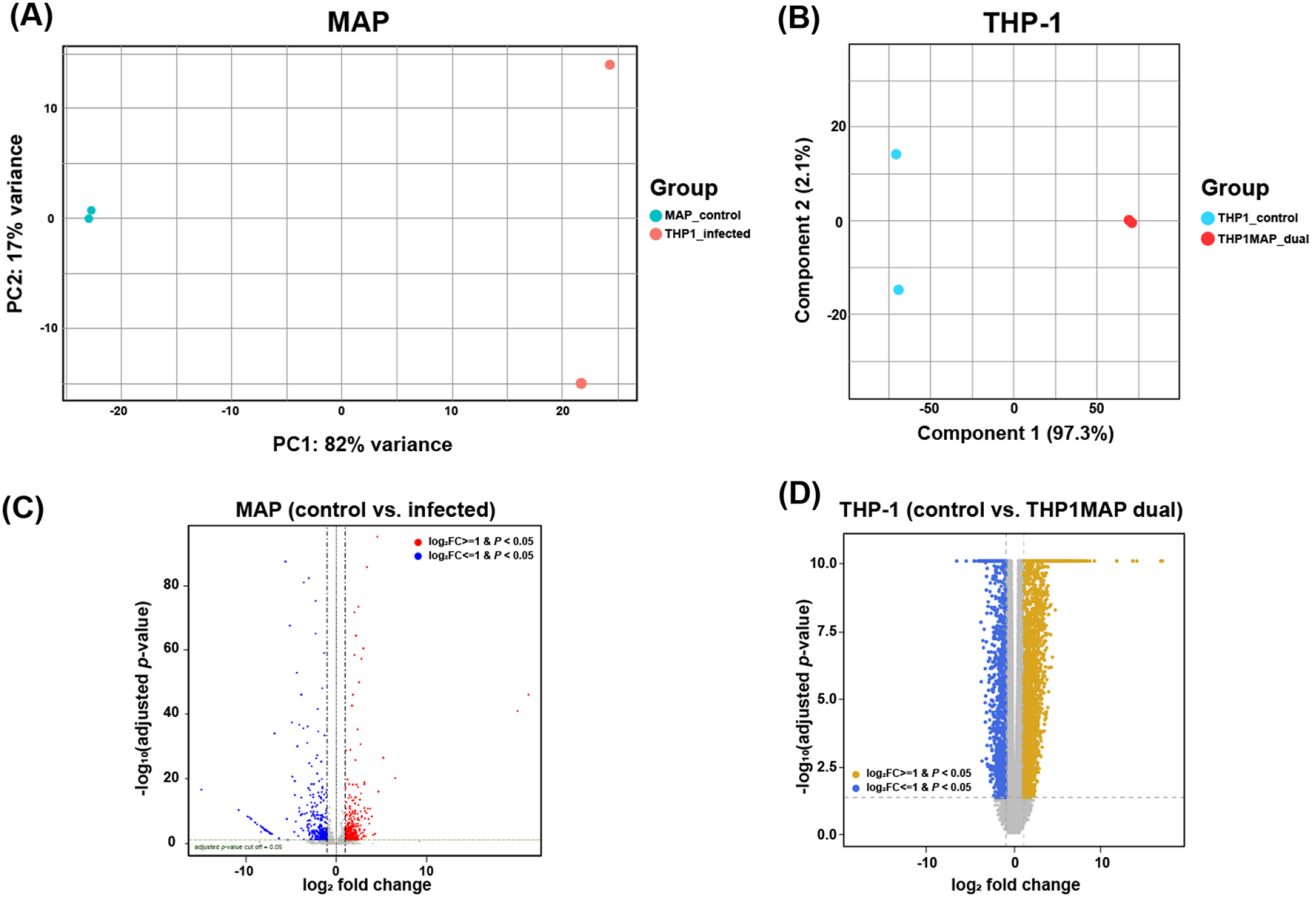
**Analysis of similarity between samples of gene expression patterns.** The variance of the sequencing data was described in a multidimensional scaling (MDS) plot comparing samples from infected and noninfected control of MAP (**A**) and THP-1 cells (**B**). Differentially expressed genes (DEGs) in infection-associated MAP (**C**) and THP-1 cells (**D**) are described in the volcano plot.

To obtain a comprehensive view of the interaction between host macrophages and MAP upon initial infection, dual RNA-seq analysis was performed 3 h post-infection to snapshot the transcriptomic profiles of both organisms. To confirm the internalization of MAP into THP-1 cells, quantitative real-time PCR was performed with the *sigA* gene, a housekeeping gene of MAP, using RNA samples before subjecting to dual RNA-seq analysis. When compared to RNA extracted from broth cultured MAP (equivalent number of infected cells), total RNA for dual RNA-seq contained 5.6-fold less in number on average, suggesting that approximately 17.8% of MAP were internalized into THP-1 (Additional file 1). Since the RNA portion of the bacteria infected with THP-1 was very small, the read count required for data interpretation of the bacteria was obtained by increasing the sequencing depth. The obtained sequencing data were mapped to the human and MAP K-10 genomes and used for subsequent analysis. The mapping rates of duplicates of MAP K-10 to the reference genome were 0.42% and 0.54%, and the absolute read counts were 1 099 954 and 1 431 536, respectively. The mapping rates for duplicates of THP-1 were 98.1% and 98.2%, respectively. Using the sequenced data, downstream analysis was performed by combining the sequencing data of broth-cultured MAP and noninfected THP-1 cells as a control for each gene expression profile. Multivariate analysis showed that the infection time had an impact on the global gene expression profiles of MAP K-10 and THP-1 cells, even though the profiles showed slight differences between replicates (Figures 1A and B). As a result of DEG analysis, the number of upregulated and downregulated genes in the intracellular MAP was 334 and 339, respectively (cutoff: |log_2_FC|≥ 1 and adjusted *p* value < 0.05) (Figure 1C). The numbers of upregulated and downregulated genes in THP-1 cells infected with MAP were 4092 and 1758, respectively (|log_2_FC|≥ 1 and adjusted *p* value < 0.05) (Figure 1D).

### mRNA repertoire of host and MAP

#### Functional enrichment analysis for MAP DEGs

**Figure 2:**
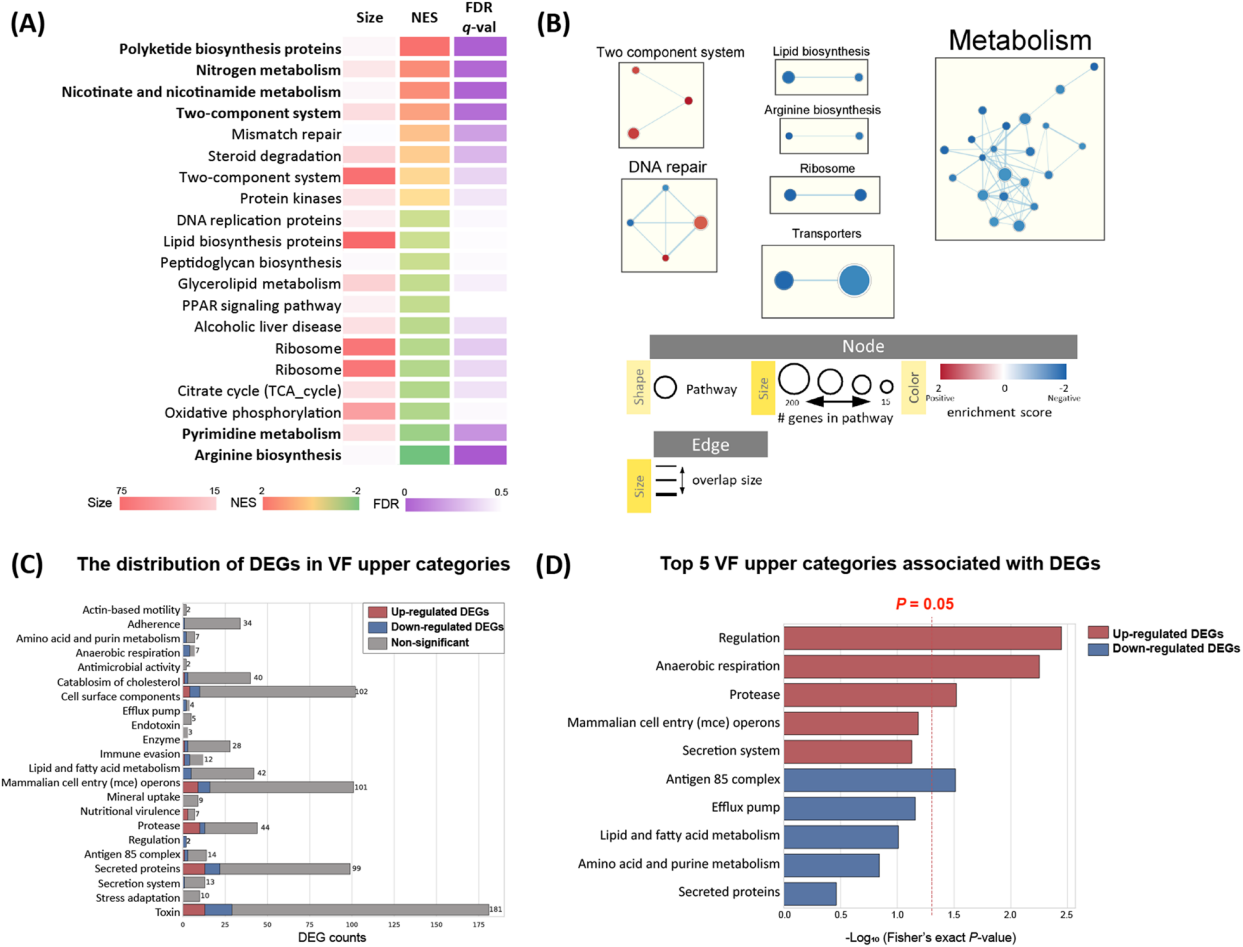
**Functional analysis of genes differentially expressed in intracellular MAP.** **A** Top 20 KEGG pathways listed according to the FDR value. Enrichment analysis was performed using the GSEA tool with customized KEGG gene sets (level C). **B** Network analysis of enriched pathways by EnrichmentMAP. Enriched pathways (*p* < 0.5) in intracellular MAP generated by the GSEA tool were exported to Cytoscape and grouped by the EnrichmentMAP application (red: positive-NES, blue: negative-NES). **C** The distribution of differentially expressed genes (DEGs) in the virulence factor upper categories (gray: nonsignificantly differed VFG-annotated loci, red: upregulated DEGs, blue: downregulated DEGs). **D** Top 5 upper categories associated with DEGs (red: upregulated DEGs, blue: downregulated DEGs). A vertical red dotted line indicates the threshold of the *p* value (*p* = 0.05).

For the functional analysis, enrichment analysis of DEGs was performed by the GSEA tool using the KEGG pathway database. A gene list of MAP corresponding to level C of the KEGG pathway database was listed and used for the GSEA. The enriched pathways were rearranged into the top 20 pathways according to the FDR value (Figure 2A) or grouped by network analysis using the “EnrichmentMAP” application from Cytoscape software (Figure 2B). As a result of the analysis, the upregulated genes were mainly enriched in pathways such as “Polyketide biosynthesis proteins”, “Nicotinate and nicotinamide metabolism”, “Nitrogen metabolism”, and “Two-component system”, whereas the pathways predicted to have the most inhibited function were “Arginine biosynthesis” and “Pyrimidine metabolism”. Network analysis of the enriched pathways showed that the “two-component system” and “DNA repair” related pathways were commonly upregulated, whereas many “Metabolism” related pathways, including “Lipid biosynthesis” and “Arginine biosynthesis”, were downregulated. The findings of the analysis suggest that MAP changes its metabolic system to adapt to the intracellular environment after infection of macrophages and it turns on the expression of pathogenic genes, such as activation of the secretion system.

#### Infection-specific VFs in the MAP genome

To investigate genetic features contributing to the pathogenicity of MAP, all locus tags in the MAP genome were subjected to BLASTp analysis against the open-source database VFDB to annotate virulence factors. Among the 28 856 VFGs in VFDB, 768 VFGs were assigned to the MAP genome (Additional file 2). Out of 673 total DEGs identified by bacterial transcriptome profiling upon infection, 123 DEGs (upregulated, 60; downregulated, 63) were also annotated (Figure 2C).

Subsequently, all VFG-annotated loci were collapsed into their functional categories to determine the overall effects of infection on virulence-related gene expression (Additional file 2). The VFGs were assigned to 43 categories, and 24 upper categories were constructed through additional curation. To obtain overrepresented categories by MAP infection, the distribution of DEGs in VFG-annotated loci was investigated (Figure 2C). On this basis, out of 24 upper categories, three upper categories were significantly associated with upregulated DEGs when assessed by Fisher’s exact test as follows: “Regulation”, *p* < 0.01; “Anaerobic respiration”, *p* < 0.01; and “Protease”, *p* < 0.03 (Figure 2D). Interestingly, several genes in the category “Regulation” encoding two component systems (TCS), such as phoP-phoR (MAP_RS01315, *phoP*; MAP_RS01320 and MAP_RS05080, *phoR*), were overexpressed. Likewise, four loci for “Anaerobic respiration” (MAP_RS10685, *narK2*; MAP_RS13350, *narI*; MAP_RS13360, *narH*; and MAP_RS13365, *narG*) and two loci for “Protease” (MAP_RS02300 and MAP_RS09345, *mpa*; MAP_RS04650, *htrA/degP*) were upregulated, thereby contributing to their functional category being significantly enriched. Meanwhile, in the case of downregulated DEGs, the “Antigen 85 complex” (*p* = 0.03) was the only upper category showing statistical significance (Figure 2D). Both genes (MAP_RS01085, *fbpA*; MAP_RS08185, *fbpB*) categorized in that category showed decreased expression.

From the perspective of individual genes, several DEGs were observed only in specific categories, although their upper categories were not significantly enriched. A gene categorized into “Macrophage inducible gene” (MAP_RS02410, *mig-5*) was the only example of an upregulated DEG. Meanwhile, the DEGs categorized as “adherence” (MAP_RS08850, *flmH*), “Amino acid and purine metabolism” (MAP_RS09970, *glnA1*; MAP_RS15490, *leuD*), “Efflux pump” (MAP_RS08120, *farB*; MAP_RS14920, *farB*), “Immune invasion” (MAP_RS10150, *acpXL*), “Magnesium uptake” (MAP_RS07770, *mgtC*), “Manganese uptake” (MAP_RS17400, *ctpC*), “*mce* operons” (MAP_RS18510, *mce*1B; MAP_RS18515, *mce*1C; MAP_RS18520, *mce*1D; MAP_RS18525, *mce*1E; and MAP_RS18530, *mce*1F), “Phagosome arrest” (MAP_RS11540, *ndk*), and “Stress adaptation” (MAP_RS08480, *kat*) were the only downregulated DEGs or nonsignificantly changed genes.

Several upper categories, such as “Catabolism of cholesterol” (up:1, down:2), “Cell surface component” (up:4, down:6), “Immune evasion” (up:1, down:2), “Lipid and fatty acid metabolism” (up:1, down:1), “Mineral uptake” (up:9, down:7), “Regulation” (up:10, down:3), “Secreted proteins” (up:1, down:2), “Secretion system” (up:13, down:9), and “Unclassification” (up:13, down:16), contained both upregulated and downregulated genes.

#### Functional enrichment analysis for THP-1 DEGs

Host responses according to MAP infection in macrophages were predicted by functional enrichment analysis using the GSEA tool with DEGs from RNA-seq data. The KEGG pathway database from MSigDB (v7.4) was used for the enrichment analysis. Among the 163 enriched gene sets, 86 and 77 were upregulated and downregulated gene sets, respectively. However, when sorted with FDR < 25%, the number of upregulated gene sets was significantly reduced, resulting in 8 and 56 up and down, respectively. Therefore, a major response to MAP infection could be predicted as an overall functional suppression.

**Figure 3:**
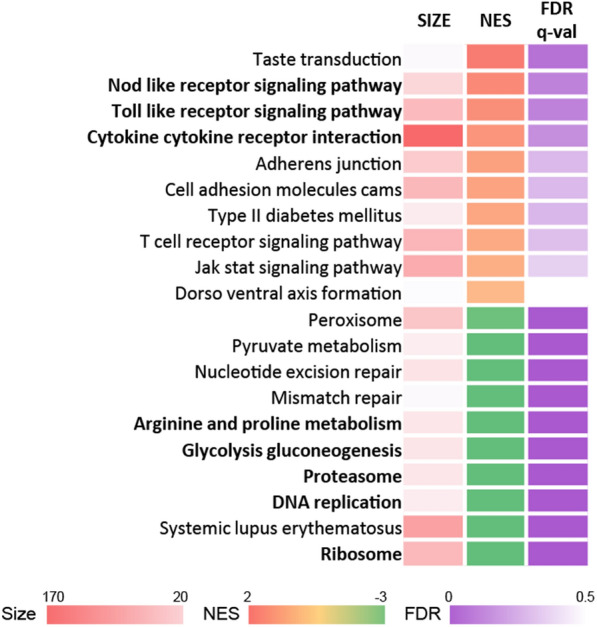
**Functional analysis of genes differentially expressed in MAP-infected THP-1 cells.** The top 20 (top 10 upregulated and top 10 downregulated) KEGG pathways are listed according to the FDR value.

The top 20 pathways predicted to have significance were created with 10 upregulated and 10 downregulated KEGG pathways in order of the lowest FDR values (Figure 3). Among the pathways with positive nominal enrichment score (NES) values, gene sets related to the innate immune response, such as the “Nod like receptor signaling pathway”, “Toll like receptor signaling pathway”, and “Cytokine cytokine receptor interaction”, were enriched. Pathways with negative normalized enrichment scores were mainly related to metabolism, and pathways such as “Ribosome”, “DNA replication”, and “Proteasome” were found to be downregulated in common. Because ribosome biogenesis is essential for cell growth [25], the predicted suppression of ribosome biosynthesis along with DNA replication indicates arrested cell growth and proliferation.

Interestingly, the “Glycolysis/gluconeogenesis” pathway was downregulated, indicating suppression of glycolysis and activation of gluconeogenesis. Among the genes consisting of this pathway, the *FBP1* gene expression was significantly upregulated while *PFKM* and *PFKP* were significantly downregulated (Additional file 3). Because proinflammatory macrophages utilize glycolysis to meet ATP requirements for phagocytosis and activation [26, 27], MAP infection seems to alter the classical activation. Expression of regulatory cytokine genes such as *IL10*, *TGFB1* and *TGFB2* were upregulated along with the upregulation of proinflammatory cytokine genes encoding TNF-α, IL-1β, and IL-23 (Additional file 3).

In particular, a trend of downregulation of the gene set related to “Arginine and proline metabolism” was observed, which is considered another interesting change considering the correlation between arginine utilization and NO production. Downregulation of arginine metabolism is expected to have a strong correlation with the observed substantial downregulation of *NOS2* gene expression. However, upregulation of the *ARG1* gene was not observed. Since downregulation of the gene set related to “Arginine biosynthesis” was also observed in MAP, the possibility of arginine depletion in the intracellular environment could be considered, and this is expected to be one of the key factors in the initial interactions during host–pathogen infection. Taken together, it is likely that MAP alters the activation and innate killing responses in THP-1 cells by inducing changes in energy metabolism and metabolic processes of amino acids such as arginine.

### Host-induced bacterial stress responses

**Figure 4:**
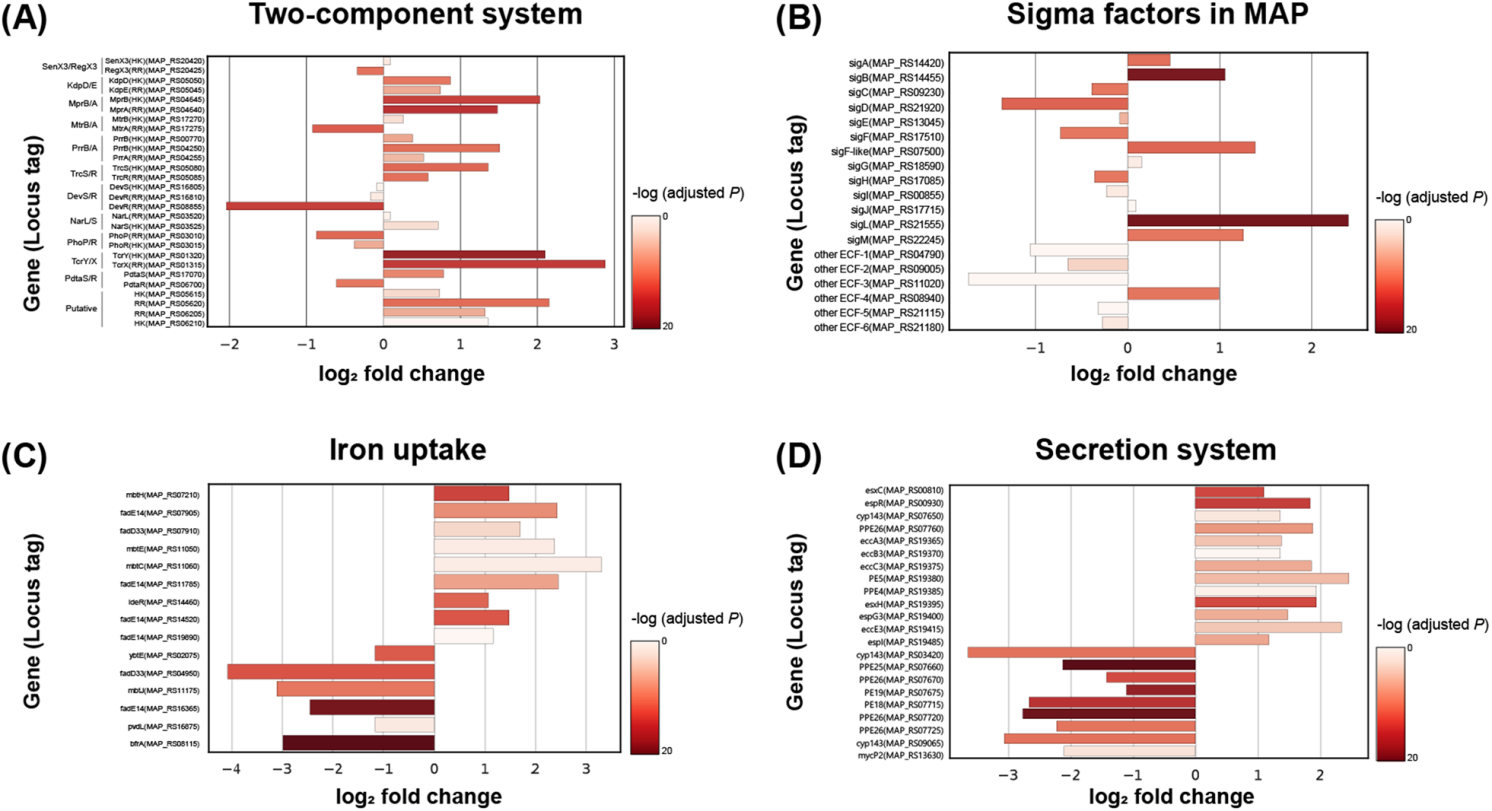
**Gene expression signatures of principal virulence factors in intracellular MAP.** Relative expression levels of genes involved in the two-component system (**A**), sigma factor (**B**), iron uptake (**C**), and the type VII secretion system (**D**).

Both KEGG pathway enrichment and VFDB annotation showed that regulatory-related genes were commonly enriched. Seven out of 10 upregulated genes and one out of three downregulated genes classified as “Regulation” in VFDB were classified as TCS in the KEGG pathway (Figures 2A and C). Four of the genes that did not belong to the TCS were sigma factors, and the other gene that was downregulated was *whiB3* (MAP_RS21910). Among the TCSs, *mprA* (MAP_RS04640) and *mprB* (MAP_RS04645) were significantly upregulated in intracellular MAP (Figure 4A). The mprAB system is known to respond to various environmental stresses and is necessary for persistent infection of *M. tuberculosis* (Mtb) in vivo [28]. *mprAB* was induced by in vivo growth of Mtb and infection in an artificial granuloma model [29, 30]. Another TCS whose expression increased in this study was *tcrX* (MAP_RS01315) and *tcrY* (MAP_RS01320). Although the function of *tcrXY* has not been studied much, the *tcrXY* knockout strain of Mtb showed hypervirulence in severe combined immunodeficient (SCID) mice [31]. There is also a report that it was upregulated under low pH conditions or low-iron conditions [32].

Among the sigma factors, *sigL* (MAP_RS21555) was the most upregulated (Figure 4B), which is consistent with the phenomenon observed during macrophage infection in a previous study [33, 34]. In a study using a *sigL* deletion mutant, it was found that *sigL* is important for acquiring resistance to stress conditions, particularly oxidative stress and stresses damaging the mycobacterial cell wall, and is essential for survival in macrophages [35]. It is known that *sigL* of Mtb regulates the gene expression involved in polyketide-lipid synthesis and genes encoding membrane-associated proteins related to posttranslational protein modification [36]. In our DEG data, several genes assigned to the *sigL* regulon (MAP_2941c (MAP_RS15050) and *mpt53* (MAP_RS15055)) and “Polyketide biosynthesis protein” (MAP_2642 (MAP_RS13465), MAP_2643 (MAP_RS13470), and MAP_3742 (MAP_RS19180)) showed significant upregulation of expression (Additional file 2).

Because iron is essential for bacterial physiological processes, iron deprivation is considered to be one of the major environmental stress conditions affecting MAP inside macrophages. Among the upregulated genes in the VFDB annotation, a number of genes were involved in iron uptake (Figures 2C and 4C). In addition, upregulation of a gene involved in mycobactin synthesis (the *mbt* gene) was observed in the “Polyketide synthesis protein” pathway, enriched by KEGG pathway analysis with the lowest FDR value (Figure 2A). Because the expression of *mbt* is up-regulated when the intracellular iron ion concentration is low to synthesize mycobactin, a siderophore of mycobacteria [37], it is predicted that MAP experiences iron deficiency inside THP-1 cells. In addition, an intracellular Fe storage gene, bacterioferritin A (*bfrA*, MAP_RS08115) [37], was also downregulated, which further supports a state of iron deficiency in MAP cells (Figure 4C). Specifically, the gene sets *mbtA* to *mbtH* (MAP_RS11035 to MAP_RS11075) corresponding to mbt-1 and *fadE14* (MAP_RS07905) and *fadD33* (MAP_RS07910) corresponding to mbt-2 were upregulated. In addition, VFDB annotation included several putative acyl-CoA dehydrogenases classified as “Iron uptake” based on a BLAST search, but their function is unknown in MAP.

Among the DEGs annotated by VFDB classification, genes classified as “Secretion system” accounted for the largest number (Figure 2C). Most of the genes annotated to this classification were the Type VII secretion system (T7SS). Among the secretion systems, a significant increase in the expression of most ESX-3 system-related genes and a significant decrease in the expression of ESX-5 system-related genes were observed (Figure 4D). Overall upregulation of the *esx-3* locus was observed. Interestingly, the expression level of the structural gene of ESX-5 did not show a significant difference compared to broth-cultured MAP. Instead, changes in the expression of PE/PPE proteins (PE19, PPE25, and PPE26) that seemed to be secreted through the system were observed (Figure 4D). From the BLAST analysis, six genes (MAP_RS07670, MAP_RS07720, MAP_RS07725, MAP_RS07740, MAP_RS07755, and MAP_RS07760) were annotated to PPE26, and two genes (MAP_RS07660 and MAP_RS07735) were annotated to PPE25. Among the six PPE26 genes, three were downregulated, one was upregulated, and the other two were not significantly changed. Among the genes annotated with PPE25, MAP_RS07660 was downregulated, and MAP_RS07735 showed no significant change. PE19 (MAP_RS07675) was significantly downregulated during infection.

### Manipulation of nitrogen-associated metabolism

**Figure 5:**
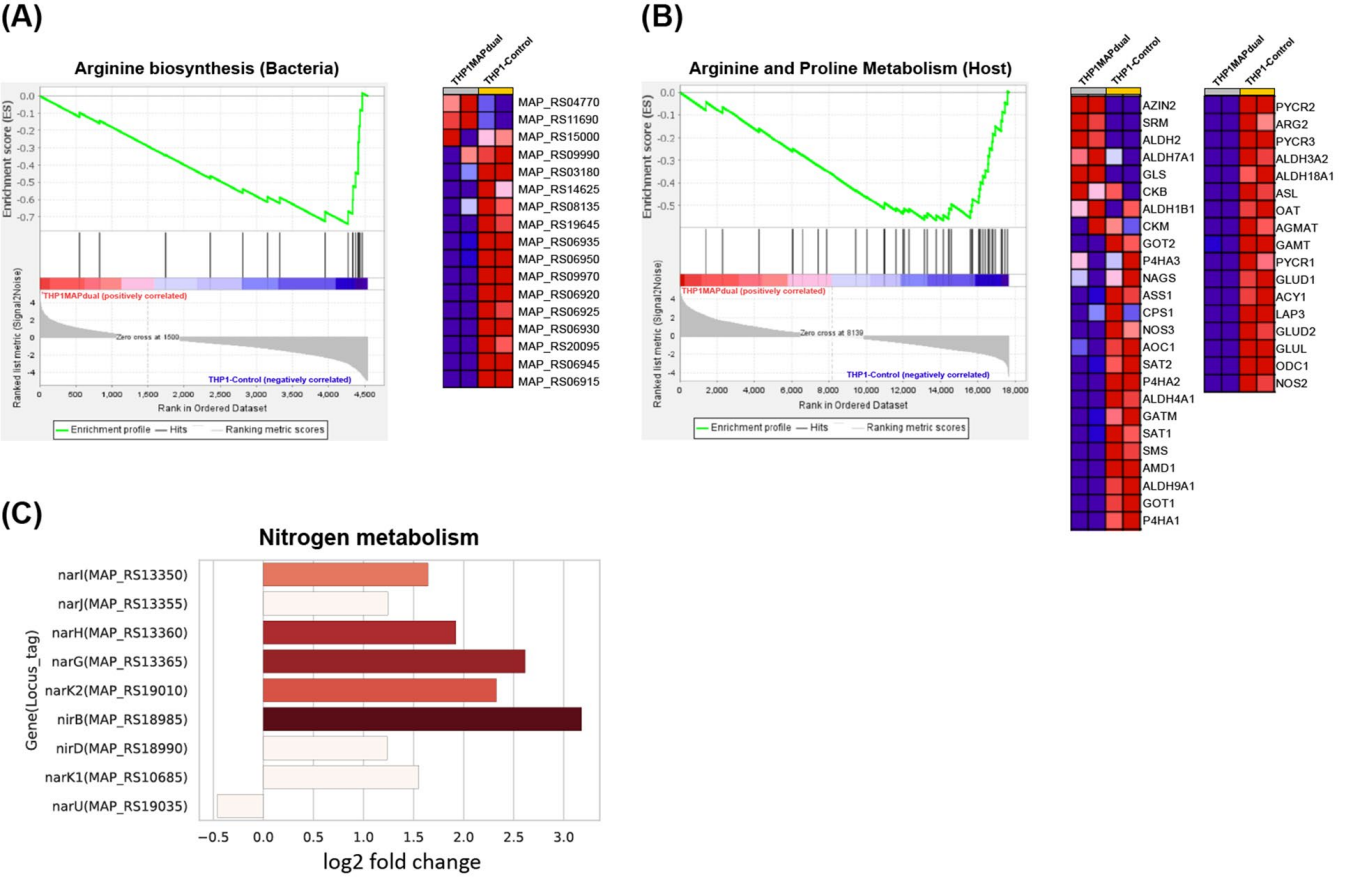
**Gene expression signatures associated with metabolic processes in hosts and pathogens.** **A** GSEA showing a negative enrichment score (ES) in the arginine biosynthesis pathway and the expression profile of related genes in MAP. **B** GSEA showing negative ES against host arginine and proline metabolism and the expression profile of related genes. **C** Relative expression of genes associated with nitrogen metabolism in MAP.

Both host and bacteria showed significant changes in genes associated with metabolism (Figures 2A, B and 3). In particular, arginine-related pathways were enriched in both. “Arginine biosynthesis” of the KEGG pathway included the production of ornithine from glutamate and the synthesis of arginine through the urea cycle. Most of the MAP genes annotated in this pathway were downregulated, and the *arcA* gene (MAP_RS04770) encoding arginine deiminase was slightly upregulated (Figure 5A). Genes downregulated in the arginine biosynthesis pathway included *argB* (MAP_RS06925), *argC* (MAP_RS06915), *argD* (MAP_RS06930), *argF* (MAP_RS06935), *argG* (MAP_RS06945), *argH* (MAP_RS06950), and *argJ* (MAP_RS06920). Meanwhile, host arginine and proline metabolism-related gene sets were downregulated (Figure 5B). This pathway included genes that are involved in catabolic reactions of arginine (*ARG1, ARG2, AZIN2, GATM*, and *NOS* genes). In particular, the changes observed in the host in this study were a significant downregulation of the *NOS2* gene, which encodes inducible nitric oxide synthase (iNOS) (Figure 5B).

As a result of KEGG pathway enrichment, it was predicted that nitrogen metabolism of MAP was upregulated (Figure 2A). Among the gene sets related to nitrogen metabolism, the nitrate reductase *narGHJI* was also annotated by VFDB analysis as “Anaerobic respiration” (Figures 2D and 5C). The upregulation of the expression of these genes in intracellular MAP compared to broth-cultured MAP appears to be related to anaerobic respiration. Changes in the expression of these gene sets are linked to pathogenesis in mycobacteria [38]. It could be an alternative energy-acquiring mechanism in the absence of oxygen by reducing nitrate into nitrite when MAP is exposed to a hypoxic environment in phagosomes. In the process of anaerobic respiration, energy is obtained through reducing NO_3_^−^ [39]. MAP seems to convert nitrite into ammonia, an energy-consuming process, by inducing the *nirB* (MAP_RS18985) and *nirD* (MAP_RS18990) genes rather than exporting nitrite in the form of nitric oxide (Figure 5C). Similar to this result, a significant increase in the expression of *nirBD* was also identified in an in vitro hypoxic dormancy model of Mtb, and the survival rate of the *nirBD* deletion mutant was significantly reduced compared to that of the wild-type strain [38]. Regarding the nitrate reduction mechanism, it was previously claimed that both *narGHJI* and *nirBD* play a role in nitrate assimilation as well as anaerobic respiration [40]. When nitrate was supplemented as the sole nitrogen source, the *narG* mutant strain did not grow, nor did the *nirB* mutant grow when nitrite was the only nitrogen source. From these results, the authors concluded that *narGHJI* and *nirBD* play vital roles in the nitrogen assimilation essential for amino acid synthesis by converting nitrate into ammonia. Taken together, the upregulation of both *narGHJI* and *nirBD* could be explained in two ways. One is the use of nitrate as an energy source under anaerobic conditions. Nitrite, a potentially toxic molecule, accumulates during this process and is cleared by energy-demanding reduction. The second is the assimilation of nitrogen. Within macrophages, nitrate can be supplied continuously [38, 41]. One piece of evidence for assimilatory nitrate reduction is the upregulation of the *narK2* (MAP_RS19010) gene, which plays a role in nitrate uptake (Figure 5C).

## Discussion

In this study, the complex host–pathogen interactions between the pathogen MAP and the host macrophage THP-1 were investigated using a dual RNA-seq approach. Within macrophages, MAP is exposed to a variety of stressful environments, including acidic pH, oxidative stress, hypoxia, and nutrient deprivation [42]. Mycobacteria have various mechanisms to respond to this environment. For example, TCS is one of the well-known signaling systems involved in the response of bacteria to environmental changes [43]. TCS recognizes several environmental stimuli and initiates an adaptive transcriptional program through a phosphoryl transfer reaction. Mtb has 12 paired TCSs in addition to two orphan HK genes and five orphan RR genes [44]. *mprAB* is a relatively well-studied TCS in mycobacteria. Functionally, *mprAB* is known to be induced in a *sigE*-dependent manner under nutrient starvation conditions and exposure to low concentrations of SDS [45]. In a study using a m*prA* deletion mutant, 141 genes were regulated by the *mprAB* system (≥ 1.8-fold above), probably because they directly regulate the transcription of global regulators such as sigma factor [46]. Several studies have confirmed that *mprAB* directly regulates the transcription of genes such as *acr2* and *pepD* as well as *sigB* and *sigE*, which are stress-responsive sigma factors [46–48]. Based on genomic analysis of MAP, it is estimated that this bacterium has 14 complete TCSs [49].

In this study, intracellular MAP showed upregulation of *mprA, mprB*, and *sigB* (MAP_RS14455) but not *sigE* (MAP_RS13045) (Figures 4A, B). This is an interesting result that no increase in *sigE* expression was observed, since an increase in *sigE* expression was a more significant change in the *mprA* overexpression condition for Mtb when external stresses, such as SDS exposure, alkaline pH, and Triton X-100, were applied [46]. In addition, it was reported that upregulation of *mprA* genes under SDS exposure was partially dependent on *sigE* [45]. The expression of *mprAB* and *sigE* are dependent on each other and they form a positive feedback loop [50]. Therefore, it is possible that the 3 h time point was too early to observe the upregulation of both genes. Another possibility is that the expression of *sigE* is regulated by other factors because sigma factors are regulated by complex networks in terms of homeostasis. Collectively, it is suggested that MAP experiences cell envelope stress in macrophages and upregulates *mprAB* to survive within that environmental condition.

Several MAP genes involved in iron depletion, particularly those regulated by *ideR*, show changes in expression when infecting host cells. It was previously reported that genes for mycobactin synthesis were the representative gene set that was upregulated under iron depletion. According to the arguments accepted thus far, however, MAP cannot synthesize mycobactin due to the truncation of the *mbtA* gene, so exogenous mycobactin J is required for it to grow in vitro [49, 51]. However, it has not been functionally validated that genetic truncation of the *mbtA* gene implies a complete loss of mycobactin synthesis [52]. Therefore, additional research is necessary to determine whether the increased expression of the *mbt* genes observed in our study can actually translate functional mycobactin in the intracellular environment. Investigating whether the putative acyl-CoA dehydrogenase genes annotated by VFDB classification in our study play a role in iron uptake might be another interesting perspective.

Among the five T7SSs of mycobacteria identified from Mtb (ESX-1 to ESX-5), MAP is known to have four of these loci, ESX-2 to ESX-5 [53]. T7SS has been studied as a key virulence factor of Mtb. In particular, the deletion of the ESX-1 system has been the most studied since it is the largest genetic event that differentiates pathogenic Mtb from the nonvirulent *M. bovis* strain bacille Calmette–Guérin (BCG) [54]. Therefore, changes in the expression of T7SS might be one of the significant indicators of pathogenic interactions between the host and MAP because this system is a potential pathway for secreting virulence factors. In our study, upregulation of ESX-3 genes was identified along with slight upregulation of the *mbt* genes, as mentioned above. This observation is consistent with a previous study in which an increase in the expression of the ESX-3 and *mbt* genes was observed under in vitro iron limiting conditions [55]. The main roles of the ESX-3 system known to date are the release of binding factors from the siderophore for extracellular iron acquisition and the transport of the bound siderophore into the intracellular area [56–58]. According to these two supportive facts, it is likely that MAP experiences iron deficiency inside macrophages. However, phenotypic confirmation is needed for this virulence gene secretion system because the role of ESX-3 system in siderophore transportation is only proved in Mtb. The virulence role of ESX-3 was confirmed through an infection experiment with a deletion mutant for the esx-3 region in Mtb [58]. The authors observed attenuation of virulence upon infection with a deletion mutant strain of the *esx-3* genes or genes encoding a protein secreted by the ESX-3 system. In particular, the iron-independent virulence role of *esxH*, which was also found to be upregulated in this study, suggests that further investigation is needed to identify the underlying mechanism of the virulence role of the ESX-3 system in MAP pathogenesis.

Another T7SS gene annotated by VFDB was ESX-5-related genes. ESX-5 is involved in the secretion of the majority of PE/PPE family proteins [59, 60]. As PE/PPE family proteins are one of the major components of the mycobacterial cell wall, they are considered to be closely related to antigenicity as well as responses to cell wall stress. A Mtb mutant strain (*ΔPstA1*) that is hypersensitive to oxygen and detergent stress showed overexpression of PE19, and deletion of *pe19* in that strain suppressed hypersensitivity to stress [61]. In this context, the downregulation of PE19 expression observed in this study might be a potential resistance strategy against stress inside macrophages. Although several genes annotated with PPE25 or PPE26 showed different expression patterns, both of the corresponding genes in the ESX-5 region (MAP_RS07660 and MAP_RS07670) were downregulated. PPE25 is a protein exposed on the surface of *Mycobacterium avium* and has been reported to be associated with pathogenicity [53]. PPE26 has been suggested to have a role as a TLR2 agonist in a study using Mtb [62].

Meanwhile, multiple metabolic processes for nutrient utilization were also significantly changed. A study using transposon mutagenesis of MAP demonstrated a decreased invasion rate in MDBK cells when the *arcA* gene was deleted [63]. Therefore, upregulation of the *arcA* gene is assumed to be necessary for effective invasion of THP-1 cells. The upregulation of the *arcA* gene and the downregulation of the *arg* gene set required for other arginine biosynthesis pathways are common changes to suppress the production of l-arginine. The *ΔargB* and *ΔargF* mutant strains of Mtb resulted in a loss of viability without l-arginine supplementation in vitro [64]. The authors also identified cell death following ROS-mediated damage in arginine auxotroph strains. In line with these results, a decrease in arginine biosynthesis during the initial stage of the MAP infection could be considered one of the defensive mechanisms of the host.

One of the major host metabolic changes was an explosive increase in the transcription of NADH dehydrogenase genes (*MT-ND1* to *MT-ND6*) in mitochondria, related to mitochondrial ROS generation along with upregulation of the *SOD2* gene (Additional file 3). However, in terms of maintaining homeostasis, downregulation of the arginine biosynthesis system suggests the possibility that the required l-arginine is being supplied from outside the cell rather than via activation of the corresponding pathway. Several pathogenic bacteria, such as mycobacteria or salmonella*,* can utilize arginine supplemented from outside the cell [65]. Upregulation of the arginine biosynthesis-related genes of Mtb in the early stage of infection was previously observed under normal conditions [64], which is the opposite of that observed in this study.

Another change observed in the host in this study was a significant downregulation of the *NOS2* gene (Figure 5B). Here, we suggest the possibility that the downregulation tendency of “Arginine and proline metabolism” enriched in the KEGG pathway of the host is related to the downregulation of *NOS2* encoding iNOS. Lack of l-arginine, which is essential for NO synthesis in macrophages, causes downregulation of the *NOS2* gene and is regulated by the *ARG1* gene that converts l-arginine to l-ornithine [66]. Although significant upregulation of the *ARG1* gene was not observed in this study, the overall trend of downregulation of arginine metabolism-related pathways, including significant downregulation of the *ARG2* gene, might indicate that cellular arginine levels are generally kept low in macrophages. One interesting change was that the *AZIN2* gene encoding arginine decarboxylase, which metabolizes l-arginine into agmatine, was significantly upregulated (Figure 5B). Since agmatine has antioxidant properties in macrophages [67], it seems to be a mechanism used to protect cells from excessive ROS generated from mitochondria. Collectively, arginine metabolism-related changes in both the host and MAP might be considered a pathogenic mechanism in terms of metabolic interactions involving inhibition of NO production.

In view of the host–pathogen interaction point, it is likely that the major response during early infection is excessive ROS production from mitochondria, which shifts the gene expression profiles of the bacteria, especially regarding oxidative stress and cell wall stress. For example, the gene expression of stress-responsive TCSs (*mprAB*, *tcrXY*) is upregulated and it regulates downstream sigma factors (*sigB*, *sigL*). Genes encoding polyketide synthase are upregulated in response to *sigL*. Another cell wall-associated protein, PE19, which is secreted by the ESX-5 system and is downregulated, might reduce the permeability of the cell wall to protect against cell wall stress. Notably, it is inferred that MAP adapts to hypoxic conditions during infection via anaerobic respiration, mainly utilizing host-derived nitrate. Moreover, metabolic changes regarding arginine in both host and pathogen may alter the production of NO, which is the major bactericidal mechanism in early infection, suggesting that arginine uptake-related genes in MAP are important virulence factors.

All of these inferences are based on the functions of genes that were not empirically demonstrated but only annotated by sequence similarity against open-source databases. Thus, studies (e.g., knockout-rescue studies) to investigate the function of these differentially expressed genes and their importance during infection by MAP are necessary. However, our dual RNA-seq data can provide novel insights by identifying broad gene expression profiles with higher resolution, especially for MAP, thereby understanding host–pathogen interactions more systematically.

## Supplementary Information

**Additional file 1.** **Quantitative real-time PCR analysis for investigating MAP internalization into THP-1 cells.** *sigA* gene, a housekeeping gene of MAP was amplified to estimate the number of MAP cells in RNA samples. (A) Amplification plot. (B) Confirmation of *sigA*-specific amplification through the melt curve analysis. (C) Estimation of relative number of MAP cells from the delta-Ct values of each sample.

**Additional file 2.** **Overall information about gene expression and VFDB annotation for each locus tag.**

**Additional file 3.** **Overall information about differentially expressed genes in THP-1 cells.**

## Data Availability

All datasets used in the RNA-seq transcriptomic analysis are available at Gene Expression Omnibus (GEO) under accession number GSE199476.

## References

[1] Naser SA, Sagramsingh SR, Naser AS, Thanigachalam S (2014). *Mycobacterium avium* subspecies *paratuberculosis* causes Crohn’s disease in some inflammatory bowel disease patients. World J Gastroenterol.

[2] Sechi LA, Dow CT (2015). *Mycobacterium avium* ss. *paratuberculosis* zoonosis the hundred year war—beyond Crohn’s disease. Front Immunol.

[3] Kuehnel MP, Goethe R, Habermann A, Mueller E, Rohde M, Griffiths G, Valentin-Weigand P (2001). Characterization of the intracellular survival of *Mycobacterium avium* ssp. *paratuberculosis*: phagosomal pH and fusogenicity in J774 macrophages compared with other mycobacteria. Cell Microbiol.

[4] Sweeney RW (2011). Pathogenesis of paratuberculosis. Vet Clin North Am Food Anim Pract.

[5] Borrmann E, Mobius P, Diller R, Kohler H (2011). Divergent cytokine responses of macrophages to *Mycobacterium avium* subsp. *paratuberculosis* strains of types II and III in a standardized in vitro model. Vet Microbiol.

[6] Motiwala AS, Janagama HK, Paustian ML, Zhu X, Bannantine JP, Kapur V, Sreevatsan S (2006). Comparative transcriptional analysis of human macrophages exposed to animal and human isolates of *Mycobacterium avium* subspecies *paratuberculosis* with diverse genotypes. Infect Immun.

[7] Cua DJ, Tato CM (2010). Innate IL-17-producing cells: the sentinels of the immune system. Nat Rev Immunol.

[8] Cunha P, Vern YL, Gitton C, Germon P, Foucras G, Rainard P (2019). Expansion, isolation and first characterization of bovine Th17 lymphocytes. Sci Rep.

[9] Park HT, Park WB, Kim S, Lim JS, Nah G, Yoo HS (2021). Revealing immune responses in the *Mycobacterium avium* subsp. *paratuberculosis*-infected THP-1 cells using single cell RNA-sequencing. PLoS ONE.

[10] Arsenault RJ, Maattanen P, Daigle J, Potter A, Griebel P, Napper S (2014). From mouth to macrophage: mechanisms of innate immune subversion by *Mycobacterium avium* subsp. *paratuberculosis*. Vet Res.

[11] DeKuiper JL, Coussens PM (2019). *Mycobacterium avium* sp *paratuberculosis* (MAP) induces IL-17a production in bovine peripheral blood mononuclear cells (PBMCs) and enhances IL-23R expression in-vivo and in-vitro. Vet Immunol Immunopathol.

[12] Dudemaine PL, Fecteau G, Lessard M, Labrecque O, Roy JP, Bissonnette N (2014). Increased blood-circulating interferon-gamma, interleukin-17, and osteopontin levels in bovine paratuberculosis. J Dairy Sci.

[13] Roussey JA, Steibel JP, Coussens PM (2014). Regulatory T cell activity and signs of T cell unresponsiveness in bovine paratuberculosis. Front Vet Sci.

[14] Bannantine JP, Conde C, Bayles DO, Branger M, Biet F (2020). Genetic diversity among *Mycobacterium avium* subspecies revealed by analysis of complete genome sequences. Front Microbiol.

[15] Lim J, Park HT, Ko S, Park HE, Lee G, Kim S, Shin MK, Yoo HS, Kim D (2021). Genomic diversity of *Mycobacterium avium* subsp. *paratuberculosis*: pangenomic approach for highlighting unique genomic features with newly constructed complete genomes. Vet Res.

[16] Cloney R (2016). Microbial genetics: dual RNA-seq for host–pathogen transcriptomics. Nat Rev Genet.

[17] Bosshart H, Heinzelmann M (2016). THP-1 cells as a model for human monocytes. Ann Transl Med.

[18] Tedesco S, De Majo F, Kim J, Trenti A, Trevisi L, Fadini GP, Bolego C, Zandstra PW, Cignarella A, Vitiello L (2018). Convenience versus biological significance: are PMA-differentiated THP-1 cells a reliable substitute for blood-derived macrophages when studying in vitro polarization?. Front Pharmacol.

[19] Langmead B, Trapnell C, Pop M, Salzberg SL (2009). Ultrafast and memory-efficient alignment of short DNA sequences to the human genome. Genome Biol.

[20] Li H, Handsaker B, Wysoker A, Fennell T, Ruan J, Homer N, Marth G, Abecasis G, Durbin R, Genome Project Data Processing Subgroup (2009). The sequence alignment/map format and SAMtools. Bioinformatics.

[21] Love MI, Huber W, Anders S (2014). Moderated estimation of fold change and dispersion for RNA-seq data with DESeq2. Genome Biol.

[22] Liu B, Zheng D, Jin Q, Chen L, Yang J (2019). VFDB 2019: a comparative pathogenomic platform with an interactive web interface. Nucleic Acids Res.

[23] Merico D, Isserlin R, Stueker O, Emili A, Bader GD (2010). Enrichment Map: a network-based method for gene-set enrichment visualization and interpretation. PLoS ONE.

[24] Reimand J, Isserlin R, Voisin V, Kucera M, Tannus-Lopes C, Rostamianfar A, Wadi L, Meyer M, Wong J, Xu CJ, Merico D, Bader GD (2019). Pathway enrichment analysis and visualization of omics data using g:profiler, GSEA, Cytoscape and enrichmentMap. Nat Protoc.

[25] Donati G, Montanaro L, Derenzini M (2012). Ribosome biogenesis and control of cell proliferation: p53 is not alone. Cancer Res.

[26] Freemerman AJ, Johnson AR, Sacks GN, Milner JJ, Kirk EL, Troester MA, Macintyre AN, Goraksha-Hicks P, Rathmell JC, Makowski L (2014). Metabolic reprogramming of macrophages: glucose transporter 1 (GLUT1)-mediated glucose metabolism drives a proinflammatory phenotype. J Biol Chem.

[27] Fukuzumi M, Shinomiya H, Shimizu Y, Ohishi F, Utsumi S (1996). Endotoxin-induced enhancement of glucose influx into murine peritoneal macrophages via GLUT1. Infect Immun.

[28] Zahrt TC, Deretic V (2001). *Mycobacterium tuberculosis* signal transduction system required for persistent infections. Proc Natl Acad Sci U S A.

[29] Karakousis PC, Yoshimatsu T, Lamichhane G, Woolwine SC, Nuermberger EL, Grosset J, Bishai WR (2004). Dormancy phenotype displayed by extracellular *Mycobacterium tuberculosis* within artificial granulomas in mice. J Exp Med.

[30] Talaat AM, Lyons R, Howard ST, Johnston SA (2004). The temporal expression profile of *Mycobacterium tuberculosis* infection in mice. Proc Natl Acad Sci USA.

[31] Parish T, Smith DA, Kendall S, Casali N, Bancroft GJ, Stoker NG (2003). Deletion of two-component regulatory systems increases the virulence of *Mycobacterium tuberculosis*. Infect Immun.

[32] Bacon J, Dover LG, Hatch KA, Zhang Y, Gomes JM, Kendall S, Wernisch L, Stoker NG, Butcher PD, Besra GS, Marsh PD (2007). Lipid composition and transcriptional response of *Mycobacterium tuberculosis* grown under iron-limitation in continuous culture: identification of a novel wax ester. Microbiology.

[33] Cossu A, Sechi LA, Zanetti S, Rosu V (2012). Gene expression profiling of *Mycobacterium avium* subsp *paratuberculosis* in simulated multi-stress conditions and within THP-1 cells reveals a new kind of interactive intramacrophage behaviour. BMC Microbiol.

[34] Ghosh P, Wu CW, Talaat AM (2013). Key role for the alternative sigma factor, SigH, in the intracellular life of *Mycobacterium avium* subsp. paratuberculosis during macrophage stress. Infect Immun.

[35] Ghosh P, Steinberg H, Talaat AM (2014). Virulence and immunity orchestrated by the global gene regulator sigL in *Mycobacterium avium* subsp. paratuberculosis. Infect Immun.

[36] Hahn MY, Raman S, Anaya M, Husson RN (2005). The *Mycobacterium tuberculosis* extracytoplasmic-function sigma factor SigL regulates polyketide synthases and secreted or membrane proteins and is required for virulence. J Bacteriol.

[37] Janagama HK, Senthilkumar BJP, Kugadas A, Jagtap P, Higgins L, Witthuhn BA, Sreevatsan S (2010). Iron-sparing response of *Mycobacterium avium* subsp *paratuberculosis* is strain dependent. BMC Microbiol.

[38] Akhtar S, Khan A, Sohaskey CD, Jagannath C, Sarkar D (2013). Nitrite reductase NirBD is induced and plays an important role during in vitro dormancy of *Mycobacterium tuberculosis*. J Bacteriol.

[39] Jung JY, Madan-Lala R, Georgieva M, Rengarajan J, Sohaskey CD, Bange FC, Robinson CM (2013). The intracellular environment of human macrophages that produce nitric oxide promotes growth of mycobacteria. Infect Immun.

[40] Malm S, Tiffert Y, Micklinghoff J, Schultze S, Joost I, Weber I, Horst S, Ackermann B, Schmidt M, Wohlleben W, Ehlers S, Geffers R, Reuther J, Bange FC (2009). The roles of the nitrate reductase NarGHJI, the nitrite reductase NirBD and the response regulator GlnR in nitrate assimilation of *Mycobacterium tuberculosis*. Microbiology.

[41] Estrella JL, Kan-Sutton C, Gong X, Rajagopalan M, Lewis DE, Hunter RL, Eissa NT, Jagannath C (2011). A novel in vitro human macrophage model to study the persistence of *Mycobacterium tuberculosis* using vitamin D-3 and retinoic acid activated THP-1 macrophages. Front Microbiol.

[42] Park HE, Lee W, Shin MK, Shin SJ (2021). Understanding the reciprocal interplay between antibiotics and host immune system: how can we improve the anti-mycobacterial activity of current drugs to better control tuberculosis?. Front Immunol.

[43] Parish T (2014). Two-component regulatory systems of mycobacteria. Microbiol Spectr.

[44] Bhattacharya M, Das AK (2011). Inverted repeats in the promoter as an autoregulatory sequence for TcrX in *Mycobacterium tuberculosis*. Biochem Biophys Res Commun.

[45] Manganelli R, Voskuil MI, Schoolnik GK, Smith I (2001). The *Mycobacterium tuberculosis* ECF sigma factor sigmaE: role in global gene expression and survival in macrophages. Mol Microbiol.

[46] He HJ, Hovey R, Kane J, Singh V, Zahrt TC (2006). MprAB is a stress-responsive two-component system that directly regulates expression of sigma factors SigB and SigE in *Mycobacterium tuberculosis*. J Bacteriol.

[47] Pang XH, Howard ST (2007). Regulation of the alpha-crystallin gene acr2 by the MprAB two-component system of *Mycobacterium tuberculosis*. J Bacteriol.

[48] Pang XH, Vu P, Byrd TF, Ghanny S, Soteropoulos P, Mukamolova GV, Wu SP, Samten B, Howard ST (2007). Evidence for complex interactions of stress-associated regulons in an mprAB deletion mutant of *Mycobacterium tuberculosis*. Microbiology.

[49] Li L, Bannantine JP, Zhang Q, Amonsin A, May BJ, Alt D, Banerji N, Kanjilal S, Kapur V (2005). The complete genome sequence of *Mycobacterium avium* subspecies *paratuberculosis*. Proc Natl Acad Sci USA.

[50] Zorzan I, Del Favero S, Giaretta A, Manganelli R, Di Camillo B, Schenato L (2021). Mathematical modelling of SigE regulatory network reveals new insights into bistability of mycobacterial stress response. BMC Bioinform.

[51] Wang J, Moolji J, Dufort A, Staffa A, Domenech P, Reed MB, Behr MA (2015). Iron acquisition in *Mycobacterium avium* subsp. *paratuberculosis*. J Bacteriol.

[52] Lamont EA, Xu WW, Sreevatsan S (2013). Host–*Mycobacterium avium* subsp. *paratuberculosis* interactome reveals a novel iron assimilation mechanism linked to nitric oxide stress during early infection. BMC Genomics.

[53] McNamara M, Danelishvili L, Bermudez LE (2012). The *Mycobacterium avium* ESX-5 PPE protein, PPE25-MAV, interacts with an ESAT-6 family Protein, MAV_2921, and localizes to the bacterial surface. Microb Pathog.

[54] Conrad WH, Osman MM, Shanahan JK, Chu F, Takaki KK, Cameron J, Hopkinson-Woolley D, Brosch R, Ramakrishnan L (2017). Mycobacterial ESX-1 secretion system mediates host cell lysis through bacterium contact-dependent gross membrane disruptions. Proc Natl Acad Sci USA.

[55] Janagama HK, Lamont EA, George S, Bannantine JP, Xu WW, Tu ZJ, Wells SJ, Schefers J, Sreevatsan S (2010). Primary transcriptomes of *Mycobacterium avium* subsp. *paratuberculosis* reveal proprietary pathways in tissue and macrophages. BMC Genomics.

[56] Serafini A, Boldrin F, Palu G, Manganelli R (2009). Characterization of a *Mycobacterium tuberculosis* ESX-3 conditional mutant: essentiality and rescue by iron and zinc. J Bacteriol.

[57] Siegrist MS, Unnikrishnan M, McConnell MJ, Borowsky M, Cheng TY, Siddiqi N, Fortune SM, Moody DB, Rubin EJ (2009). Mycobacterial Esx-3 is required for mycobactin-mediated iron acquisition. Proc Natl Acad Sci USA.

[58] Tufariello JM, Chapman JR, Kerantzas CA, Wong KW, Vilcheze C, Jones CM, Cole LE, Tinaztepe E, Thompson V, Fenyo D, Niederweis M, Ueberheide B, Philips JA, Jacobs WR (2016). Separable roles for *Mycobacterium tuberculosis* ESX-3 effectors in iron acquisition and virulence. Proc Natl Acad Sci U S A.

[59] Abdallah AM, Verboom T, Weerdenburg EM, Gey van Pittius NC, Mahasha PW, Jimenez C, Parra M, Cadieux N, Brennan MJ, Appelmelk B, Bitter W (2009). PPE and PE_PGRS proteins of *Mycobacterium marinum* are transported via the type VII secretion system ESX-5. Mol Microbiol.

[60] Ates LS, Ummels R, Commandeur S, van der Weerd R, Sparrius M, Weerdenburg E (2015). Essential role of the ESX-5 secretion system in outer membrane permeability of pathogenic mycobacteria. PLoS Genet.

[61] Ramakrishnan P, Aagesen AM, McKinney JD, Tischler AD (2016). *Mycobacterium tuberculosis* resists stress by regulating PE19 expression. Infect Immun.

[62] Su HB, Kong C, Zhu L, Huang Q, Luo LL, Wang HH, Xu Y (2015). PPE26 induces TLR2-dependent activation of macrophages and drives Th1-type T-cell immunity by triggering the cross-talk of multiple pathways involved in the host response. Oncotarget.

[63] Alonso-Hearn M, Patel D, Danelishvili L, Meunier-Goddik L, Bermudez LE (2008). The *Mycobacterium avium* subsp *paratuberculosis* MAP3464 gene encodes an oxidoreductase involved in invasion of bovine epithelial cells through the activation of host cell Cdc42. Infect Immun.

[64] Tiwari S, van Tonder AJ, Vilcheze C, Mendes V, Thomas SE, Malek A, Chen B, Chen M, Kim J, Blundell TL, Parkhill J, Weinrick B, Berney M, Jacobs WR (2018). Arginine-deprivation-induced oxidative damage sterilizes *Mycobacterium tuberculosis*. Proc Natl Acad Sci USA.

[65] Gogoi M, Datey A, Wilson KT, Chakravortty D (2016). Dual role of arginine metabolism in establishing pathogenesis. Curr Opin Microbiol.

[66] Bronte V, Murray PJ (2015). Understanding local macrophage phenotypes in disease: modulating macrophage function to treat cancer. Nat Med.

[67] Chai JS, Luo L, Hou FY, Fan X, Yu J, Ma W, Tang WQ, Yang X, Zhu JY, Kang WY, Yan J, Liang HP (2016). Agmatine reduces lipopolysaccharide-mediated oxidant response via activating PI3K/Akt pathway and up-regulating Nrf2 and HO-1 expression in macrophages. PLoS ONE.

