# Supplementary figures and images for: Delineating transcriptional crosstalk between Mycobacterium avium subsp. paratuberculosis and human THP-1 cells at the early stage of infection via dual RNA-seq analysis
Source: Vet Res. 2022 Sep 13;53:71. doi: 10.1186/s13567-022-01089-y (PMC9469519; doi:10.1186/s13567-022-01089-y)

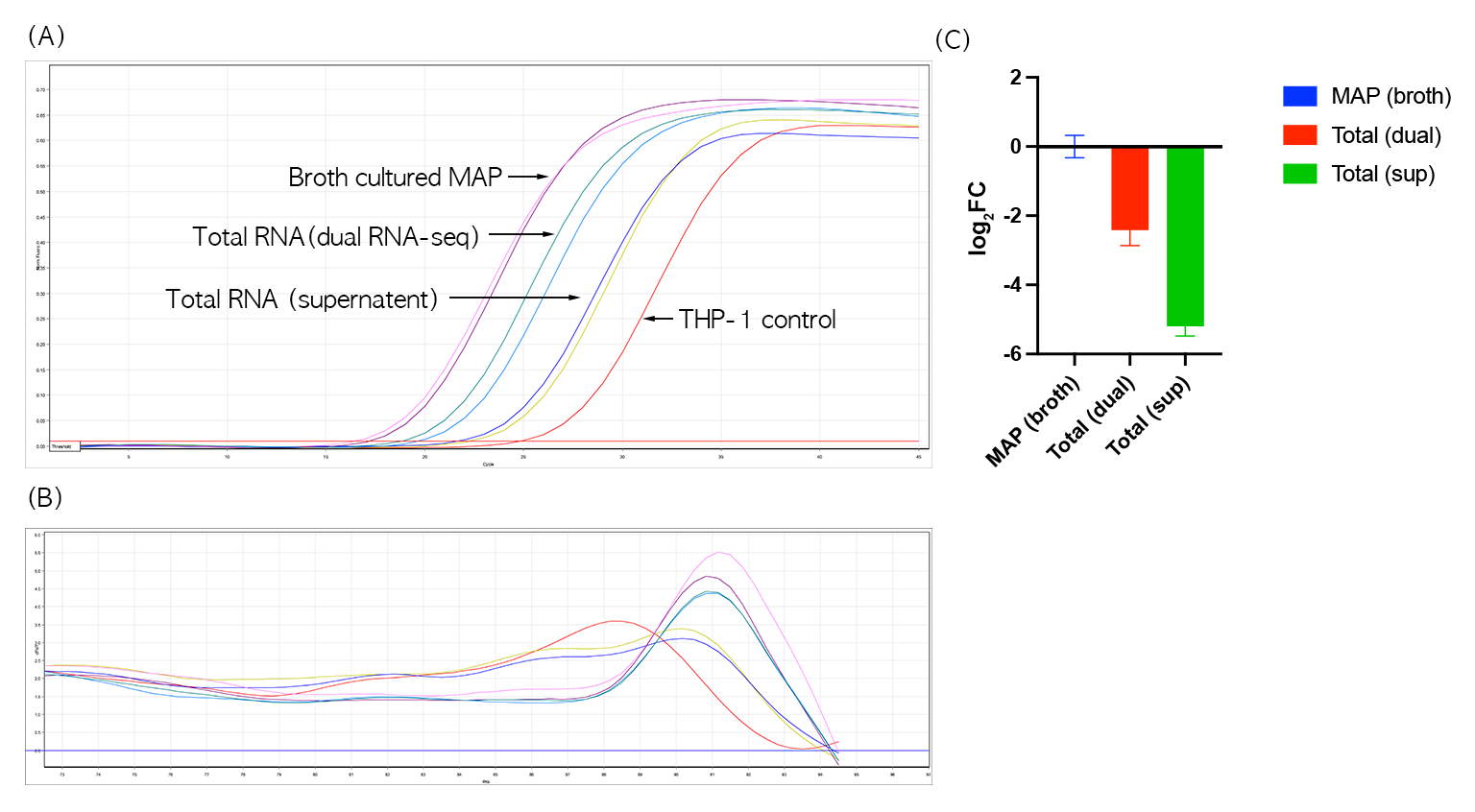

Supplement: Supplementary file 1 — Additional file 1. Quantitative real-time PCR analysis for investigating MAP internalization into THP-1 cells. sigA gene, a housekeeping gene of MAP was amplified to estimate the number of MAP cells in RNA samples. (A) Amplification plot. (B) Confirmation of sigA-specific amplification through the melt curve analysis. (C) Estimation of relative number of MAP cells from the delta-Ct values of each sample. [file 13567_2022_1089_MOESM1_ESM.tif]
